# Immune mechanism of *n*-butanol extract of *Clerodendrum bungei* against loach infected with *Aeromonas hydrophila*

**DOI:** 10.3389/fimmu.2025.1597463

**Published:** 2025-05-01

**Authors:** Ya-Jie Li, Xin-Ya Dong, Hong-Hui Li, Jin-Guo Luo, Xing-Yu Chen, Xu-Dong Zhou

**Affiliations:** ^1^ Traditional Chinese Medicine (TCM) and Ethnomedicine Innovation & Development International Laboratory, Innovative Materia Medica Research Institute, School of Pharmacy, Hunan University of Chinese Medicine, Changsha, China; ^2^ The First Hospital of Hunan University of Chinese Medicine, Hunan University of Chinese Medicine, Changsha, China

**Keywords:** *Clerodendrum bungei* Steud, *Aeromonas hydrophila*, antibacterial mechanism, immunity, Chinese medicine

## Abstract

**Introduction:**

Traditional Chinese medicines (TCMs) have a wide variety of chemical components and biological activities, which are applied in multiple fields such as medicine, agriculture and food. *Clerodendrum bungei* (*C. bungei*), known as “ChouMuDan” in Chinese, is a traditional herb belonged to the shrub of the genus *Clerodendrum* and used to treat various diseases. Previous studies indicated *C. bungei* had certain activity in antimicrobial aspects, therefore, the present study focus on exploring its antibacterial effect on loaches infected with *A. hydrophila* and the mechanism.

**Methods:**

This study first prepared the *n*-butanol extract of *C. bungei*, using relux extraction and liquid-liquid organic partition method. Then, the loach was randomly divided into two groups. After the experiment, the livers of loaches from two different groups were dissected for microRNA sequencing. The samples were sequenced in Hiseq Single-End mode to obtain the original data. The Unique Reads were aligned with the non-coding RNA sequences in the Rfam 13 database using Blast to screen out the non-coding RNAs and identify their types and numbers for further analysis of their functions and regulatory mechanisms. The Reads Count value of miRNA was calculated, and the expression data of the conserved miRNA in each sample were sorted out. Finally, seven differentially expressed miRNA that regulate immunity, were selected to verify the credibility of miRNA expression identified by sequencing.

**Results:**

Two miRNA libraries of mCK1 and mC4 were constructed, using the Hiseq Single-End mode, to study the effect of *n*-butanol extract of *Clerodendrum bungei Steud*. (BECB) on loach infected with *A. hydrophila*. Differentially expressed miRNAs were identified, among which 9 were up-regulated and 17 were down-regulated. The cluster analysis of differentially expressed miRNAs showed that the expression pattern of miRNAs changed significantly after BECB treatment. KEGG enrichment analysis was carried out on target genes, and the top 30 most significantly enriched pathways were selected by p-value. The result suggested the immune-related pathways mTOR signaling pathway and RIG-I-like receptor signaling pathway may critical for resistance to *A. hydrophila* infection in loach after BECB treatment, together with RT-qPCR results.

**Discussion:**

In this study, pathways related to carbohydrate and lipid metabolism were enriched after infection with *A. hydrophila* for 24 h, which may be because the proliferation and differentiation of immune cells require a large number of nutrients to provide energy. This indicates that pathogens did not appear in the loach until exposure to *A. hydrophila* for 24 h. After infection, both autophagy and mTOR signaling pathway are activated to promote the proliferation and differentiation of immune cells, induce the production of type I IFN and inflammatory factors, and then trigger innate and specific immunity. These findings could provide a basis for the research and development of antibacterial agents based on *C. bungei* and the application of biopesticides in aquaculture, especially in loach farming.

## 1 Introduction

Loach (*Misgurnus anguillicaudatus*) has strong adaptability, good meat quality and high nutritional value, which is a good product for nourishment and health care. It can be used for both fresh and dry, and medicinal purposes, with high economic value (1). The protein hydrolysate of loach exhibited strong antioxidant activity (2). *M. anguillicaudatus* carbohydrates (MAC) isolated from loaches could increase the production of immune substances and is expected to become a potential immune agent (3). Although loach has its own research value as a source of medicinal materials and a common species, in the cognition of most people, it exists as a food material and one of the exported aquatic products, because of their simple production and good taste. In the meantime, as the scale of its breeding and farming are also increasing year by year, the ensuing disease prevention and treatment has also attracted much attention (4). High density farming is easy to lead to immune suppression and even bacterial disease outbreak. *A. hydrophila*, as the most common human-animal-fish co-infection in freshwater farmed fish (1, 5), it is susceptible to infection not only when the immune function is low, but also when the immune function is normal (6), which not only pollutes water, but also causes economic losses to the food industry and serious harm to human health (7). Various virulence factors carried by *A. hydrophila* could cause hemorrhagic septicemia, ulcer infection and erythrosores in fish, and human infection with *A. hydrophila* can cause skin and soft tissue infections, intestinal disorders and blood diseases (6). Previous studies have found that *A. hydrophila* B11 can survive in tilapia macrophages (8), indicating that *A. hydrophila*’s survival ability is continuously strengthened and its harm is greater. Currently, antibiotics are the most commonly used treatment methods in the prevention and control of bacteria, but the improper use of antibiotics will lead to the development of drug resistance in bacteria, and the residual drugs in water will cause environmental pollution. In order to solve this problem, people have gradually turned their attention to Chinese herbal medicines or other natural substances.

It is known that natural products exhibit diverse bioactivities and have great value in agricultural applications. Chinese medicinal herbs themselves can be used not only for drug compatibility but also as inspiration for new therapeutic agents. *Clerodendrum bungei* (*C. bungei*) Steud. is the shrub of the genus *Clerodendrum* in the family Labiaceae. Its roots, stems and leaves could be used as medicinal herbs. It is one of the commonly used ethnic veterinary plants in the animal husbandry of the Yao people living in the southwestern province of China (9). Previous studies on *C. bungei* mainly focus on its chemical composition and pharmacological effects. Some of the compounds isolated from *C. bungei* can reduce the mortality of CLP-induced sepsis in mice and have strong anti-inflammatory effects *in vivo (* 10), and some have inhibitory effects on the HeLa human cervical carcinoma cell line (CCL-2) *in vitro* (11). Others can inhibit the activities of angiotensin-converting enzyme and α-glucosidase (12). The methanolic extract from *C. bungei* and its main components can be used to develop insect repellent (13), the acetone extract of the root has obvious anti-complement activity (14), and its leaf extract has hemostatic effect (15). Despite the gradual advancement in modern pharmacology and the study of its active ingredients, which have helped to address the shortcomings of *C. bungei* in contemporary scientific systems, there are still many unknown effects of this plant waiting for us to discover. Previous literatures have indicated that *C. bungei* also has a certain role in immunity (15), but the mechanism is still unknown. Therefore, we used loaches infected with *A. hydrophila* to explore the antibacterial effect and mechanism of *C. bungei*.

## 2 Materials and methods

### 2.1 Experimental materials and pretreatment

The aerial parts of *Clerodendrum bungei* Steud were collected in September 2021 at Changsha, Hunan Province (GPS coordinates: Longitude 112◦94′298″E, Latitude 28◦24′073′′N). They were extracted by reflux with 80% ethanol twice, every time 2 hours. The resulting liquid was filtered and concentrated under reduced pressure at 50°C, using a vacuum rotary evaporator, to yield the total crude extracts (2.5 kg). Subsequently, the ethanol extract was suspended with water and then submitted to liquid-liquid partition with organic solvents to afford petroleum ether, dichloromethane, ethyl acetate and *n*-butanol (504.5 g) extracts. *A. hydrophila* used for infection was donated by Wetland College of Yancheng Normal University and the number of bacteria was calculated by plate scoring method. The loach used in the experiment was purchased from Mingfang Aquatic Seafood Store, Ouhai District, Wenzhou city, Zhejiang Province. The loach was domesticated in the laboratory environment for 14 days. During the domestication period, the feed was special for loach, and fed after changing the water in the morning and evening every day.

The loach was randomly divided into two groups. Before the experiment, the loach was changed with water and fed with feed. The treatment group C4 was added with 1% feed amount of *n*-butanol extract of *Clerodendrum bungei Steud. (*BECB), and the control group CK1 was not added. *A. hydrophila* was put into the water to make the amounts of bacteria in the water reach 10^5^ CFU/mL. After 24h of infection, the liver of loach was dissected under anesthesia, quick-frozen in liquid nitrogen and stored at -80°C. Part of the liver was taken for microRNA sequencing and the rest was prepared for later use.

### 2.2 Data processing

After processing, the samples were sequenced in Hiseq Single-End mode to obtain the original data. A script developed by the company was used to filter the data, remove the splices and perform quality cutting according to sequence quality to obtain clean reads for subsequent analysis, and the filtered sequence length was more than 18nt. clean reads with sequence length between 18 and 36 nt were counted, and the identical sequences within each sample were deduplicated and the corresponding abundance of the sequences was recorded. The deduplicated sequences were called Unique Reads.

The Unique Reads were aligned with the non-coding RNA sequences in the Rfam 13 database using Blast to screen out the non-coding RNAs and identify their types and numbers for further analysis of their functions and regulatory mechanisms. The four known classes of ncRNA (rRNA, tRNA, snRNA, snoRNA) were screened by screening criteria (perfect matches or no more than two mismatches). At the same time, the sequences were aligned to the mature miRNA sequences of all plants in miRBase22 according to the screening criteria mentioned above to screen the conserved mirnas. Since Small RNA sequences are very short and can be easily matched to different fragments, there are various annotation results. The annotation results of sequences in this paper will be sorted out according to the order of known miRNA, rRNA, tRNA, snRNA and snoRNA.

### 2.3 Data analysis

According to the number of sequences aligned to the conserved miRNA, the Reads Count value of miRNA was calculated, and the expression data of the conserved miRNA in each sample were sorted out. DESeq was used to analyze differentially expressed miRNA, and the criterion was that the expression fold difference was greater than 2 and the significance was less than 0.05.

In order to further study the potential function of miRNA related to BECB improving body immunity and enhancing antibacterial effect, we used miRanda to predict target genes by using the principle of miRNA binding target sites through complementary pairing. The target gene sequence was derived from UniGene obtained by non-reference transcriptome sequencing, and the function of miRNA was inferred by enrichment analysis of target genes. We map all target genes into each Term in the GO database, count the number on each Term, and use hypergeometric distribution to calculate the significantly enriched terms against the whole genome. The number of target genes included in different levels of each KEGG pathway was counted and the metabolic pathways and signaling pathways mainly involved by target genes were determined by Rich factor and FDR value.

### 2.4 miRNA data validation

In order to verify the credibility of miRNA expression identified by sequencing, we selected 7 differentially expressed miRNA that regulate immunity. RNA was extracted by Tiangen Animal Extraction kit, and cDNA was synthesized by Vazyme miRNA Reverse transcription kit for fluorescence quantification. The detailed information of primers is shown in **Table 1**. RT-qPCR was used to check the expression of the selected miRNA in mCK1 and mC4 with β-actin as the internal reference, and the consistency with sequencing results was compared.

**Table 1:** Primers used for qRT-PCR.

miRNAs	Mature sequence	Forward primer(5.’-3’)	Adaptor primer
miR-27a-3p	UUCACAGUGGCUAAGUUCAGU	TTCACAGTGGCTAAGTTCAGT	Universal reverse Q preimer in miRNA 1^st^ strand cDNA Synthesis Kit
miR-18c	UAAGGUGCAUCUUGUGUAGU	TAAGGTGCATCTTGTGTAGT	As above
miR-459-5p	UCAGUAACAAGGAUUCAUCCUG	TCAGTAACAAGGATTCATCCTG	As above
miR-205-5p	UCCUUCAUUCCACCGGAGUCUG	TCCTTCATTCCACCGGAGTCTG	As above
miR-454b	UAGUGCAAUAUUGCUUAUAGGGUC	TAGTGCAATATTGCTTATAGGGTC	As above
miR-725-3p	UUCAGUCAUUGUUUCUGGUAGU	TTCAGTCATTGTTTCTGGTAGT	As above
miR-206-3p	UGGAAUGUAAGGAAGUGUGUGG	TGGAATGTAAGGAAGTGTGTGG	As above
β-actin		AGAGAGAAATTGTCCGTGAC	As above

## 3 Results

### 3.1 miRNA sequencing and annotation

**Figure 1:**
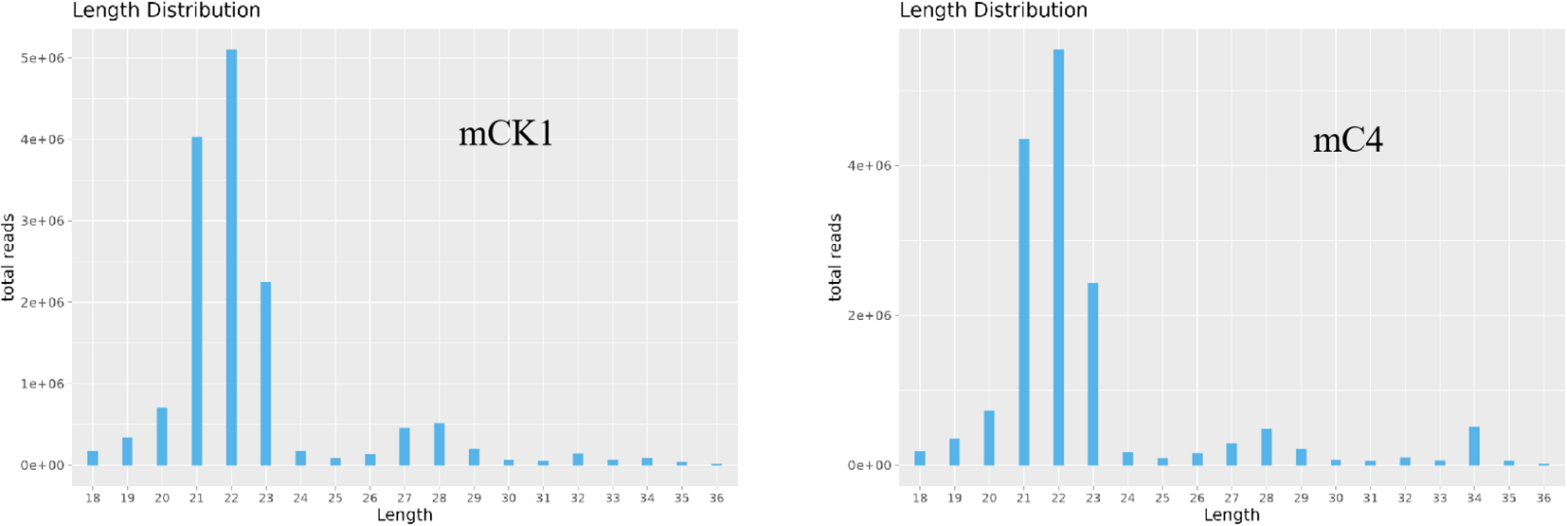
Sequence length profile of clean reads. The horizontal axis is sequence length, and the vertical axis is sequence abundance (*10000).

**Figure 2:**
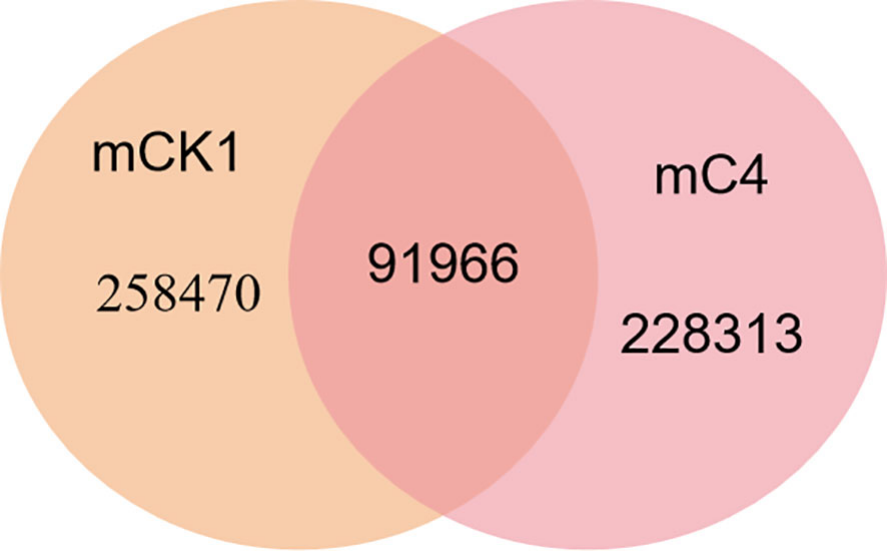
Venn plot of mCK1 versus mC4 deduplicated sequences.

**Figure 3:**
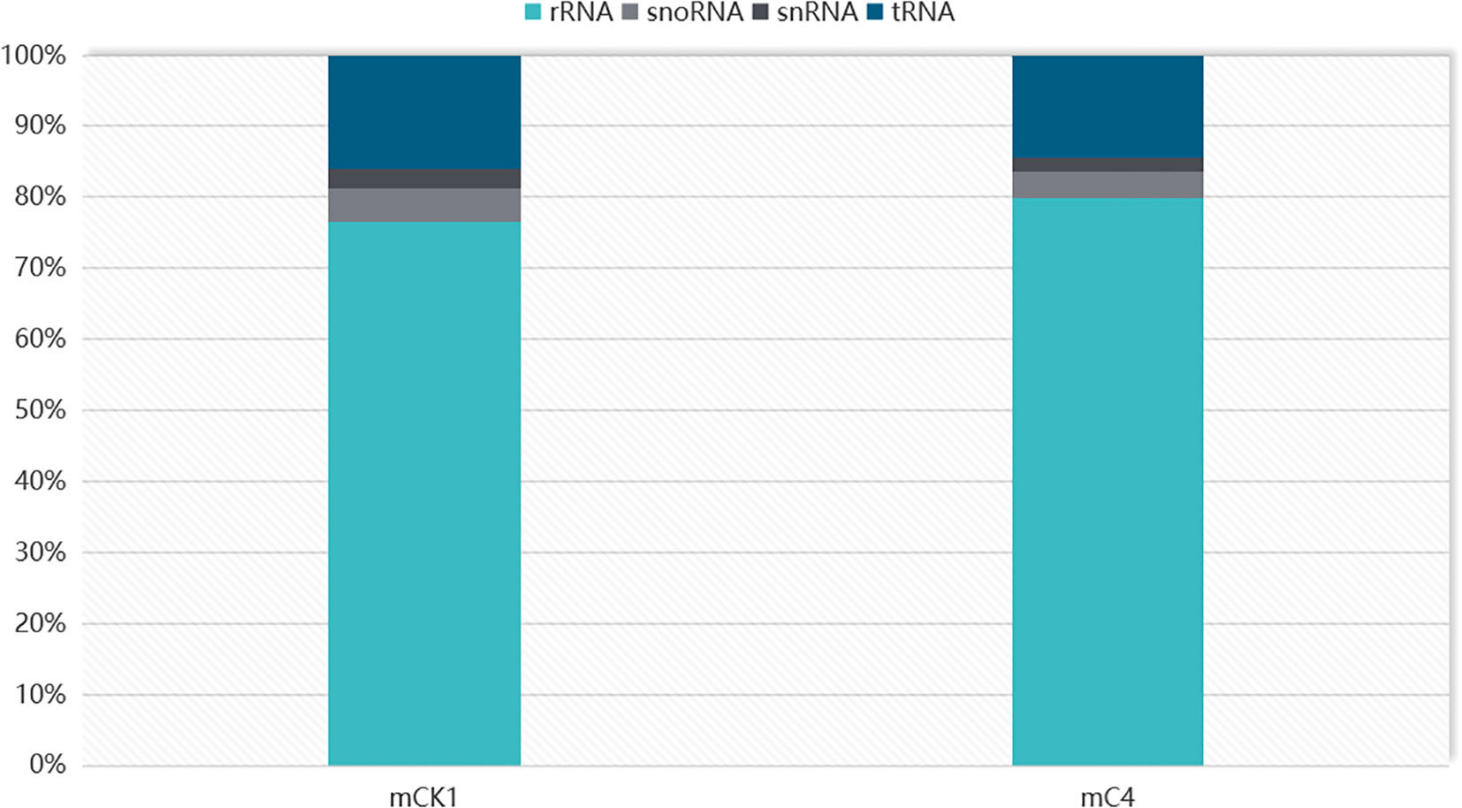
Classification of ncRNAs.

In order to study the effect of BECB on loach infected with *A. hydrophila*, two miRNA libraries of mCK1 and mC4 were constructed. Hiseq Single-End mode was used to complete the sequencing work, and 16239217 and 17919776 original data were obtained in mCK1 and mC4 respectively. After removing splice and low-quality reads, 14660513 and 15908200 clean reads were obtained, accounting for 90.28% and 88.77% of the original data respectively (**Table 2**). From the sequence length distribution, it can be seen that the sequence length of the two libraries is basically concentrated between 21 and 23 nt (**Figure 1**). After removing the repetitive sequences, there was 91966 common sequence, 258470 unique to CK1 and 228313 unique to C4 (**Figure 2**). Using Rfam database to screen non-coding RNAs, it can be seen that the unique reads obtained in mCK1 and mC4 all belong to non-coding RNAs, among which the most alignment to rRNA in mCK1 and mC4 are 72221 and 77985, respectively (**Figure 3**). In mCK1 and mC4, there were 14790 and 14418 mature miRNAs whose sequences could be matched in miR Base database, respectively, and there were 924 and 903 matched data in miR Base database, respectively.

**Table 2:** An overview of CK1 and C4 in data analysis.

	mCK1	mC4
Raw reads	16239217	17919776
Clean reads	14660513	15908200
Annotated	2250829	2552128
Unique small RNAs	94276	93310

**Figure 4:**
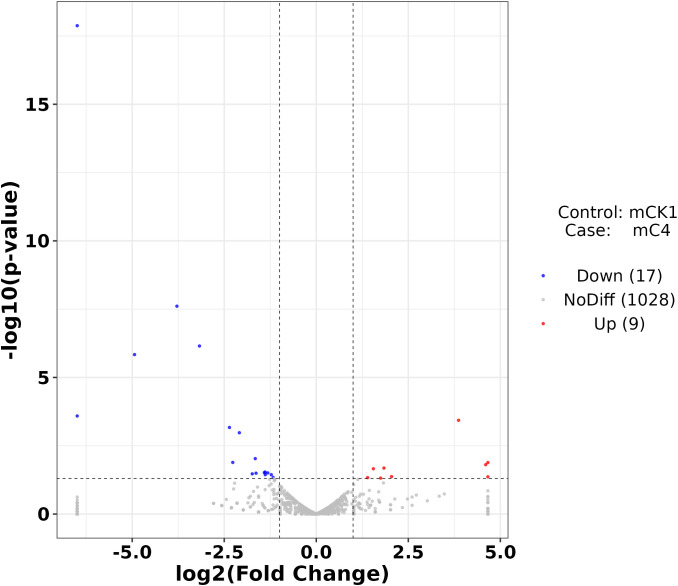
Volcano plot of mcroRNAs differentially expressed between mCK1 and mC4. Upregulation is indicated in red and downregulation in blue.

**Figure 5:**
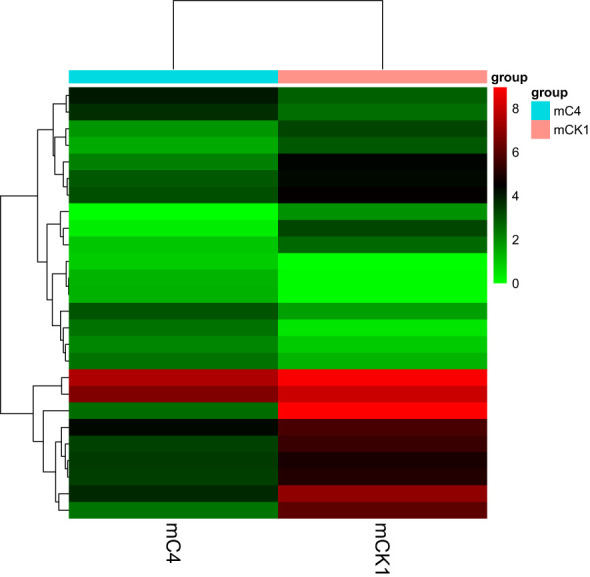
Graph of clustered expression patterns of differential microRNAs.

The number of sequences to conserved miRNA was counted, the expression of miRNA was calculated. Differentially expressed miRNAs were identified according to the screening criteria mentioned above, among which 9 were up-regulated and 17 were down-regulated (**Figure 4**). The cluster analysis of differentially expressed miRNAs showed that the expression pattern of miRNAs changed significantly after BECB treatment (**Figure 5**).

**Figure 6:**
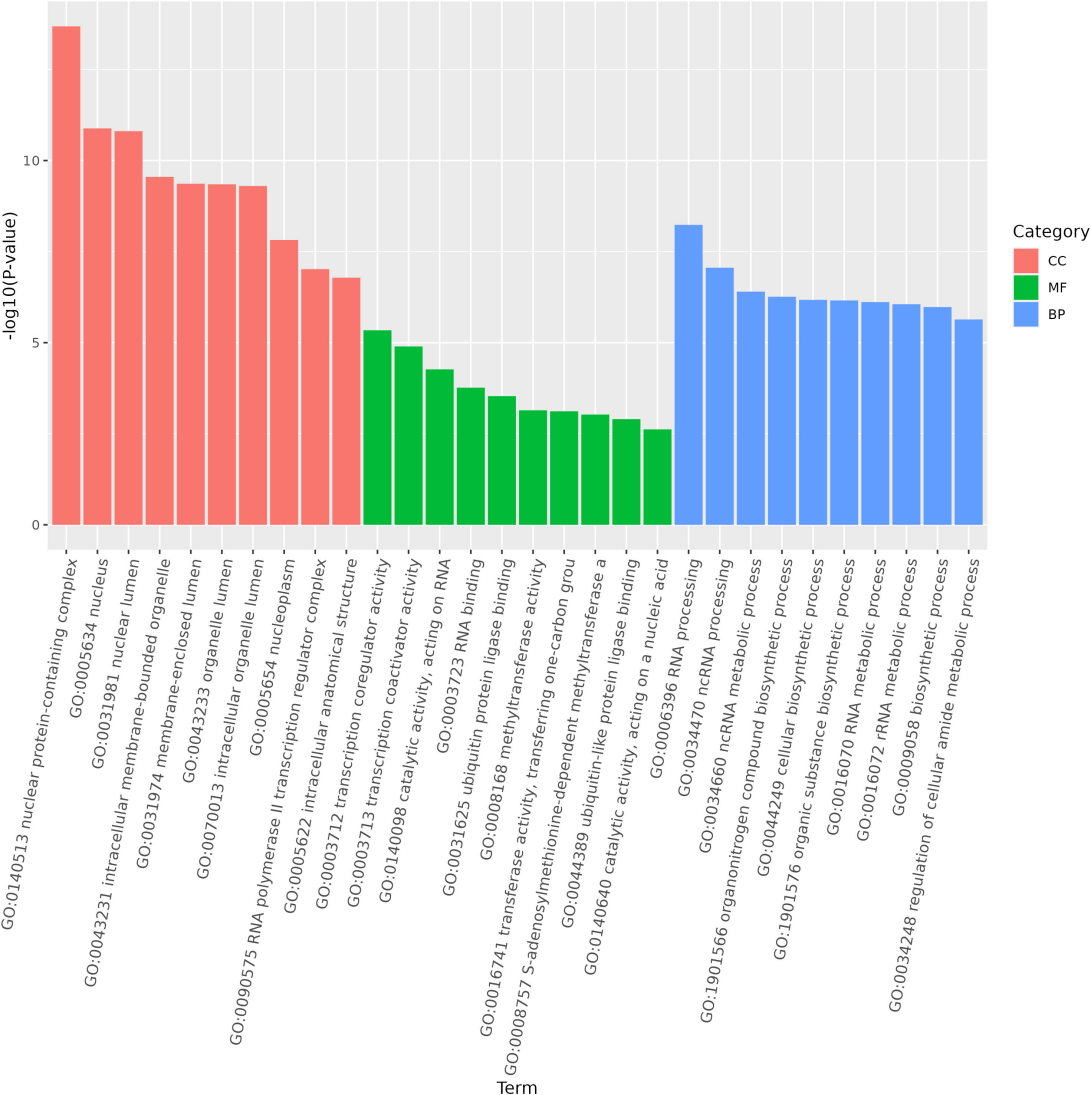
Top 10 enriched terms in each category of GO.

**Figure 7:**
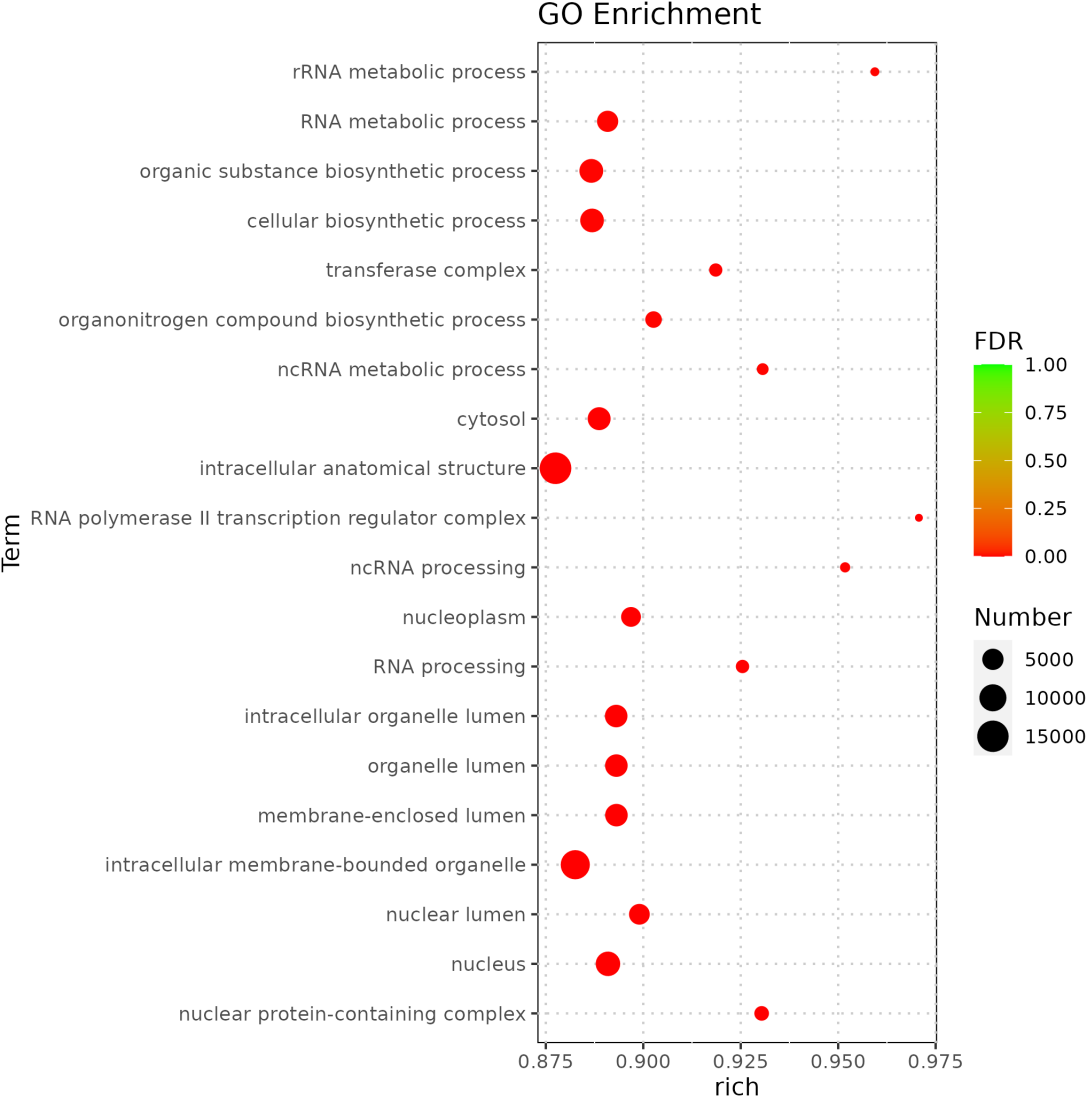
Top 20 GO enrichment of target genes regulated by differential mcroRNA.

**Figure 8:**
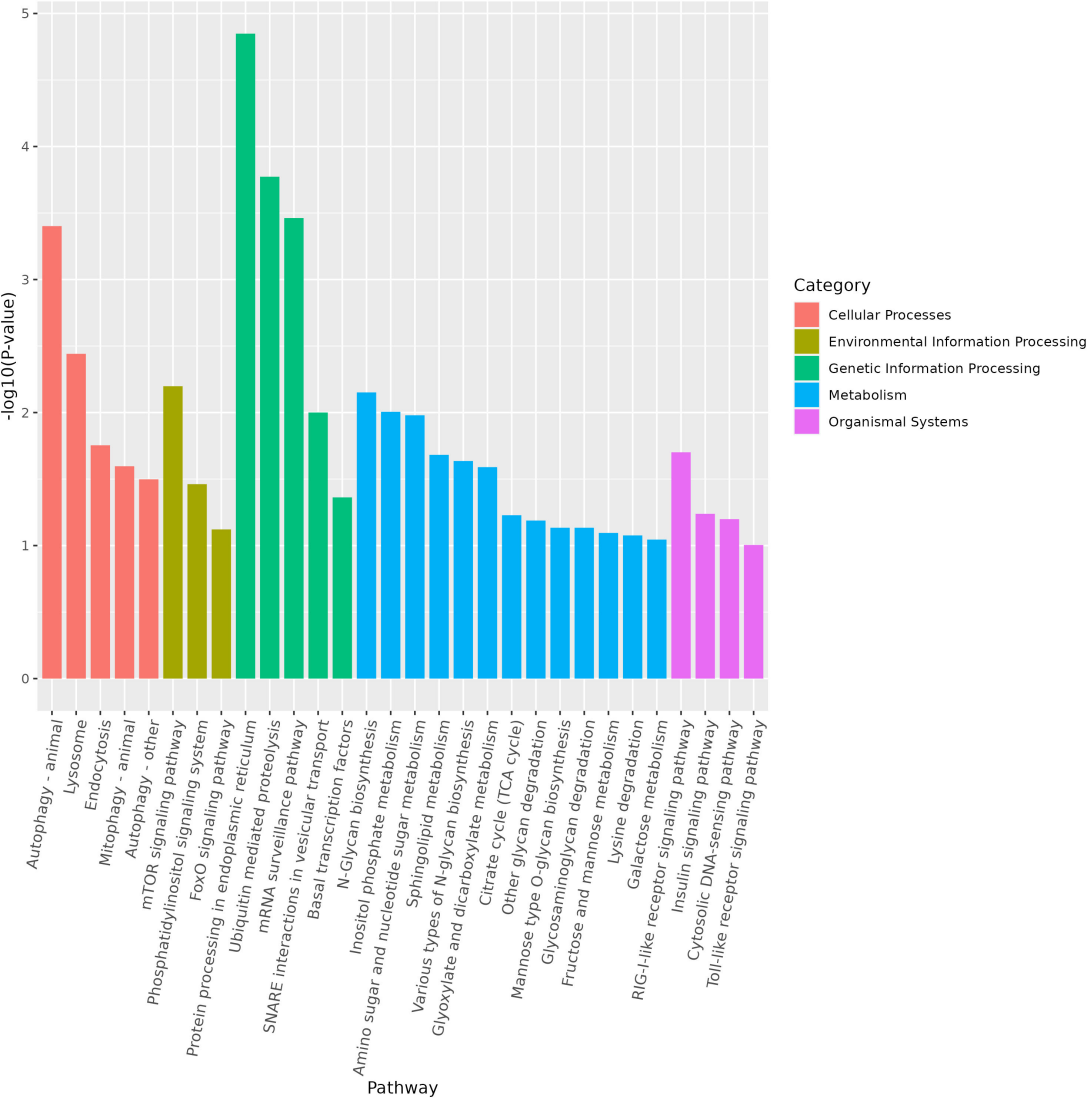
Top 30 most significantly enriched pathways in KEGG.

**Figure 9:**
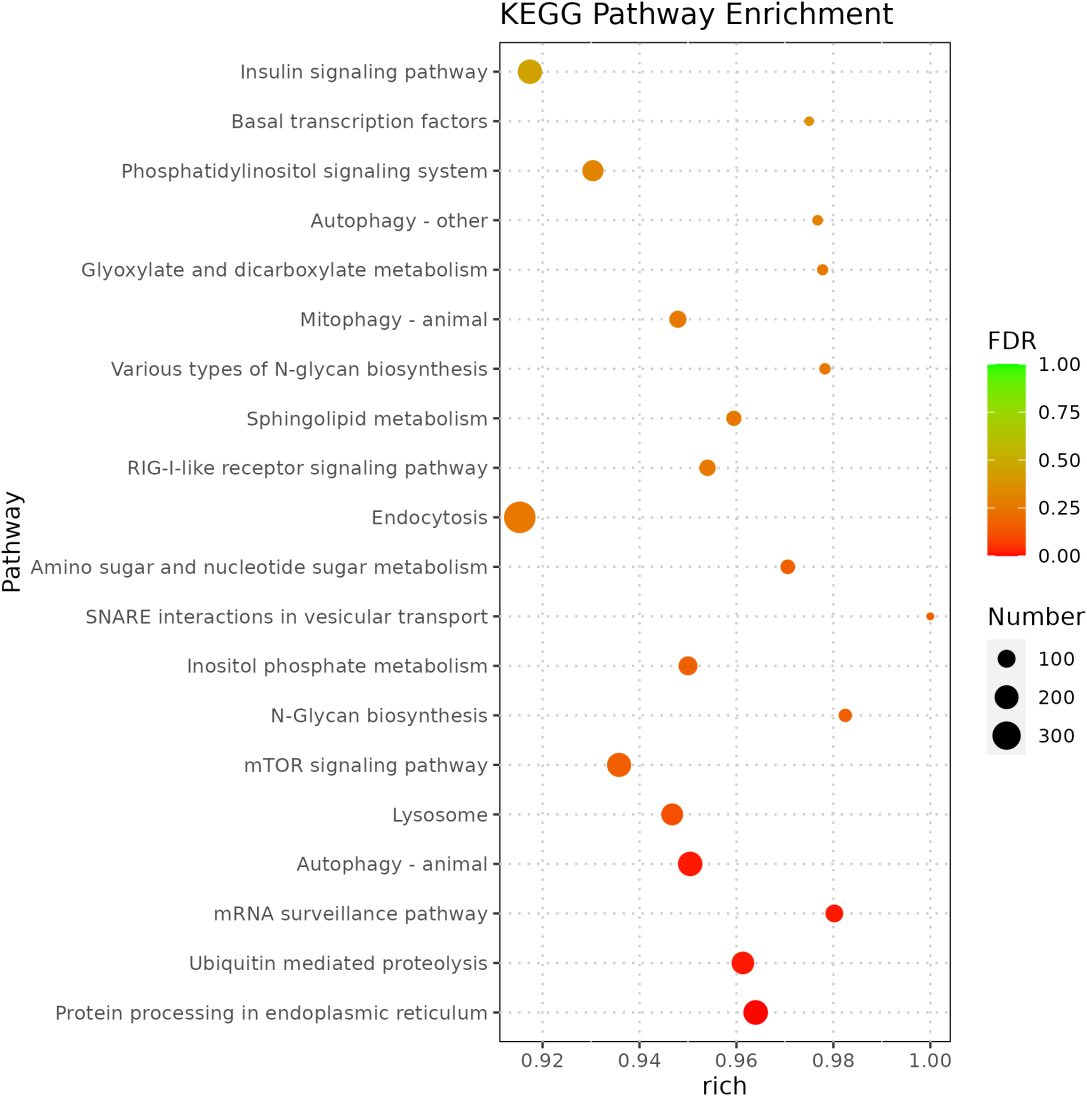
Top 20 KEGG enriched pathways for target genes regulated by differential microRNA.

miRNA is non-coding RNAs and cannot produce functional proteins. In order to infer the function of miRNAs, we predicted target genes according to the principle of base complementary pairing, and then determined the function of miRNA through enrichment analysis of target genes. From GO enrichment of the top 10 most significantly enriched terms in each category, it can be seen that the molecular functions of differential miRNAs are mainly concentrated in the Cellular Component (**Figure 6**). Therefore, most of the top 20 enriched GO terms in the classification were Cellular Component, and a few were Biological Process. Among the Cellular components, the most significant are intracellular membrane-bounded organelle and intracellular anatomical structure, the most significant ones in Biological Process are cellular biosynthetic process and organic substance biosynthetic process (**Figure 7**). KEGG enrichment analysis was carried out on target genes, and the top 30 most significantly enriched pathways were selected by p-value. Among them, the most enriched pathway was Metabolism, and the most significantly enriched pathways were Genetic information Processing and Cellular Processes (**Figure 8**). This indicated that loaches treated with BECB were still in the initial stage of infection when exposed to *A. hydrophila* for 24 h. Among the top 20 most significantly enriched pathways (**Figure 9**), the immune-related pathways mTOR signaling pathway and RIG-I-like receptor signaling pathway and Signal transduction related pathway the Phosphatidylinositol signaling system were found Enrichment of signaling systems that may be critical for resistance to *A. hydrophila* infection in loach after BECB treatment. In addition, many related to energy metabolism and Transport and catabolism may also play a role in the antimicrobial mechanism of loach.

**Figure 10:**
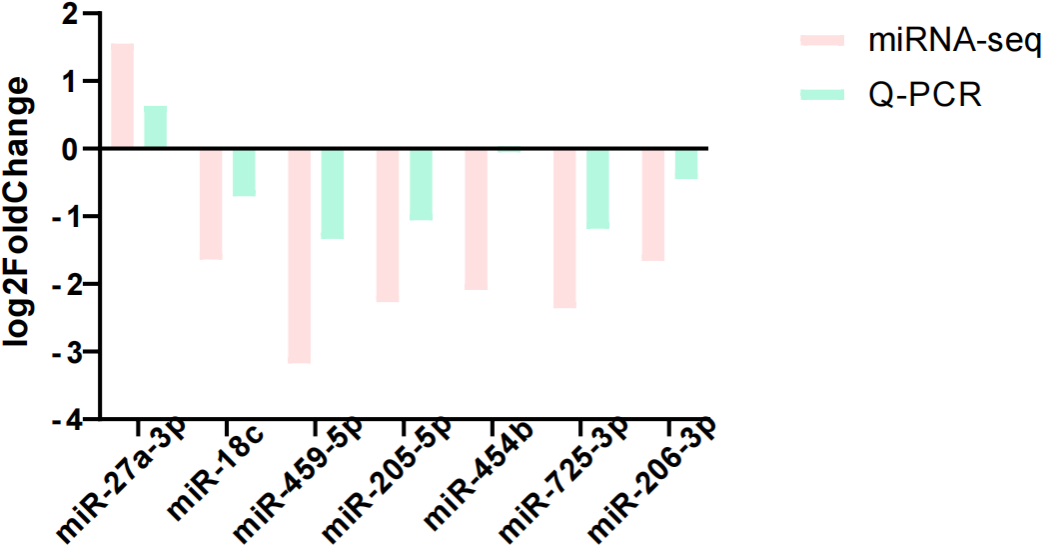
Comparison between sequencing results and qRCR results. Log^2Fold Change^ > 0 was defined as upregulation and less than 0 as downregulation.

We selected seven differentially expressed miRNAs for quantitative analysis by RT-qPCR to verify the sequencing results. From the comparison results in **Figure 10**, it can be seen that the experimental conclusion is basically the same as the sequencing results, and the sequencing data can be used for subsequent experiments.

## 4 Discussion

The innate immune system detects invading microbes through three types of pattern recognition receptors and microbial-specific nucleic acid detection, with RIG-I-like receptors (RLRs) being one of them (16). RLR has been shown to initiate and regulate antiviral immunity in many studies (17–19), but many studies have found that this pathway has a non-negligible role in tumors (20–22) and even in bacterial infections (23–25). Maybe RLR can sense not only viral RNA but also cytoplasmic DNA. Myoung Kwon Choi et al. (26) found that RIG-I and MDA5 could be used as cytosolic DNA sensors for B-DNA. However, more studies have shown that DNA from bacteria released into the cytoplasm can be used as DNA sensors through DAI and IFI16 to induce the production of type I IFN and cytokines in RLR-independent pathways (27). In addition, it has been reported that RLR can respond to bacterial infection probably because bacterial DNA activates STING (stimulator of interferon genes) and is regulated by STING to induce the production of type I IFN and activate the body’s defense mechanism to produce cytokines, thus stimulating specific immunity (28). However, the expression of these genes was not seen in this study. Perhaps the loach infected with *A. hydrophala* can activate the RIG-I-like receptor signaling pathway because of pathogenic RNA produced during bacterial infection, in which results the production of type I IFN and triggers innate immunity (29).

Autophagy is an innate immune response of cells and can interact with immune signaling pathways to promote pathogen elimination. Autophagy plays an important role in antigen recognition and presentation, development and maturation of immune cells, and promotion of lymphocyte homeostasis (30, 31). Autophagy in immune cells is related to antigen processing and presentation and T cell activity (32). Cells capture and phagocytize pathogens through autophagy and present them to effector T cells for the purpose of immune surveillance, so that cytokines and chemokines can be released to activate surrounding immune cells (33, 34). The presentation of endogenous antigens is also regulated by the autophagy pathway, and antigen presenting cells can capture and degrade intracellular antigens through autophagy (35). T lymphocytes can promote the proliferation of T cells through autophagy after antigen stimulation, which may be because it is a survival mechanism of cells (35). In the nutrient-deficient microenvironment, autophagy can provide sufficient nutrients for cells by degrading dysfunctional organelles, proteins and lipids to maintain cell homeostasis in response to metabolic stimulation (32). In this study, pathways related to carbohydrate and lipid metabolism were enriched after infection with *A. hydrophila* for 24 h, which may be because the proliferation and differentiation of immune cells require a large number of nutrients to provide energy. This indicates that pathogens did not appear in the loach until exposure to *A. hydrophila* for 24 h.

The mechanistic target of rapamycin (mTOR) protein is an atypical serine/threonine protein kinase that can regulate cell growth and metabolism, and is the node that integrates environmental factors and cellular functions (36). Over-activation of mTOR signaling leads to metabolic reprogramming, changes in cell cycle, and inhibition of apoptosis and autophagy, so mTOR immunosuppressants are used for the treatment of a variety of diseases (37–40). α-Mangostin inhibits the formation and growth of skin cancer by inhibiting PI3K/AKT/mTOR signal transduction, inducing tumor autophagy and promoting apoptosis, thus significantly reducing the incidence (41). Bajijiasu can reduce metabolic decomposition and inflammatory response of osteoarthritis (OA) *in vitro* and *in vivo*, possibly by regulating AKT/mTOR/NF-κB signaling pathway to promote autophagy (42). The activation of mTOR signaling pathway can also promote the release of proinflammatory factors and trigger inflammation (43). In addition, mTOR also has a significant regulatory ability in the maturation of antigen presenting cells, differentiation and activation of immune cells (42, 44). Immune cells can sense integration through mTOR signal transduction to promote M2 macrophage polarization, antigen presentation, marginal zone (MZ) B cell proliferation, plasma cell differentiation, etc. (45, 46). Therefore, after infection with *A. hydrophila* for 24 h, mTOR signaling pathway is activated in loach, which causes inflammation and then drives and regulates the immune response of the body. The activation of mTOR signaling pathway can inhibit autophagy, but autophagy was not inhibited in this study, which illustrate that the body is in the beginning stage of immunity. Both autophagy and mTOR signaling pathway are activated to promote the proliferation and differentiation of immune cells, induce the production of type I IFN and inflammatory factors, and then trigger innate and specific immunity. In conclusion, BECB can enhance the resistance of loach to *A. hydrophila*.

## 5 Conclusions

To study the effect of BECB on the immune mechanism of loach infected by *A. hydrophila* by microRNA sequencing. After infection, the body provides energy to cells in the way of autophagy to promote the development, differentiation and maturation of immune cells, and promotes the recognition and presentation of antigens in combination with mTOR signaling pathway to initiate and regulate innate immunity. In addition, pathogenic RNA produced during bacterial infection may be related to the activation of RIG-I-like receptor signaling pathway to produce type I IFN to trigger innate immunity. According to the analysis results, loach was in the early stage of infection 24 hours after infection, indicating that the BECB has the effect of enhancing the immunity of the body.

## Data Availability

The datasets presented in this study can be found in online repositories. The names of the repository/repositories and accession number(s) can be found below: https://www.ncbi.nlm.nih.gov/genbank/, SRR29790702.
